# Immunological mechanisms of the nucleocapsid protein in COVID-19

**DOI:** 10.1038/s41598-024-53906-3

**Published:** 2024-02-14

**Authors:** Fahime Edalat, Niloofar Khakpour, Hossein Heli, Arash Letafati, Amin Ramezani, Seyed Younes Hosseini, Afagh Moattari

**Affiliations:** 1 Department of Bacteriology and Virology, Shiraz University of Medical Sciences, Shiraz, Iran; 2 Nanomedicine and Nanobiology Research Center, Shiraz University of Medical Sciences, Shiraz, Iran; 3 Department of Virology, School of Public Health, Tehran University of Medical Sciences, Tehran, Iran; 4 Shiraz Institute for Cancer Research, School of Medicine, Shiraz University of Medical Science, Shiraz, Iran; 5 Department of Medical Biotechnology, School of Advanced Medical Sciences and Technologies, Shiraz University of Medical Sciences, Shiraz, Iran

**Keywords:** Coronavirus, Apoptotic, Cytokine storm, Nucleocapsid protein, SARS, Cell signalling, SARS-CoV-2, Viral host response, Viral pathogenesis, Viral vectors, Virus-host interactions

## Abstract

The emergence of corona virus disease 2019 (COVID-19), resulting from Severe acute respiratory syndrome coronavirus 2 (SARS-CoV-2), has left an indelible mark on a global scale, causing countless infections and fatalities. This investigation delves into the role of the SARS-CoV-2 nucleocapsid (N) protein within the HEK293 cells, shedding light on its influence over apoptosis, interferon signaling, and cytokines production. The N gene was amplified, inserted into the pAdTrack-CMV vector, and then transfected to the HEK293 cells. Changes in the expression of IRF3, IRF7, IFN-β, BAK, BAX, and BCL-2 genes were evaluated. The levels of proinflammatory cytokines of IL-6, IL-12, IL-1β, and TNF-α were also determined. The N protein exhibited an anti-apoptotic effect by modulating critical genes associated with apoptosis, including BAK, BAX, and BCL-2. This effect potentially prolonged the survival of infected cells. The N protein also played a role in immune evasion by suppressing the interferon pathway, evidenced by the downregulation of essential interferon regulatory factors of IRF3 and IRF7, and IFN-β expression. The N protein expression led to a substantial increase in the production of proinflammatory cytokines of IL-6, IL-12, IL-1β, and TNF-α. The N protein emerged as a versatile factor and was exerted over apoptosis, interferon signaling, and cytokine production. These findings carry potential implications for the development of targeted therapies to combat COVID-19 and mitigate its global health impact.

## Introduction

The coronavirus disease 2019 (COVID-19) pandemic that is caused by the severe acute respiratory syndrome coronavirus 2 (SARS-CoV-2) has had far-reaching repercussions on a global scale and is highly pathogenic and contagious^1,2^. Frequent signs of COVID-19 include fever, dry cough, exhaustion, and respiratory distress. In severe cases, individuals may experience complications including acute respiratory distress syndrome, acute lung injury, septic shock, or, in the most extreme cases, fatal outcomes^3^. The pathogenic mechanism of SARS-CoV-2 must be clarified so that effective therapeutic routes are developed^4^.

SARS-CoV-2 is a wrapped and positively oriented RNA virus with a viral genomic RNA size of ~ 30 kilobases. It belongs to the β-coronavirus family. The viral genome of SARS-CoV-2 consists of 14 operational open reading frames (ORFs)^5,6^. This virus contains two primary segments, specifically ORF1a and ORF1b, responsible for encoding 16 non-structural proteins (NSP1-NSP16). Additionally, there exist nine regions that encode nine potential accessory proteins, along with other segments accountable for encoding four structural proteins: spike (S), envelope (E), membrane (M), and nucleocapsid (N) proteins^7^. Notably, the S protein of SARS-CoV-2 possesses the ability to bind to the angiotensin-converting enzyme-2 (ACE2), which serves as its cellular receptor. This crucial interaction facilitates the entry of the virus into the cells^8^.

N protein can attach to RNA in the viral genome to form the ribonucleoprotein. The M and E proteins, meanwhile, are essential for starting the assembly process of the virus^7^. The N protein of SARS-CoV-2 has functional RNA-binding domains at both its N- and C-terminus. These domains are crucial to the viral replication process because they play a key role in encapsulating the viral genomic RNA^9^. Previous studies have unveiled several noteworthy impacts of the N protein on the host cells. It has been observed that activation of the NLRP3 inflammasome leads to cytokine storms and lung injury^10^. The N protein also impairs the synthesis of interferon beta (IFN-β) by blocking retinoic acid-inducible gene I (RIG-I) signaling through the DExD/H domain^11^. It has also been reported that the N protein has roles to increase viral replication by preventing the formation of stress granules^12^ and to suppress the antiviral immune response by controlling the activity of mitochondrial antiviral-signaling (MAVS) protein^13^.

Understanding the complex interactions between the host cells and viral proteins is critical for comprehending the pathogenesis of COVID-19^14^. Notably, proteins like B-cell lymphoma-2 (BCL-2), BCL-2 antagonist/killer (BAK), and BCL-2 antagonist X (BAX) affect apoptosis as a basic cellular process. These proteins are crucial for controlling the stability between programmed cell death and cell survival. BAK and BAX, pro-apoptotic participants of the BCL-2 family, inspire apoptosis in response to positive cell stresses, whereas BCL-2, renowned for its anti-apoptotic function, works to prevent cellular apoptosis^15^. Gaining knowledge about how SARS-CoV-2 (and specifically its N protein) affects these important apoptotic regulators might help better understand the complex host-virus interactions that contribute to the severity and spread of COVID-19. By assessing the expression levels of BCL-2, BAK, and BAX in human embryonic kidney 293 (HEK293) cells expressing the SARS-CoV-2 N gene, the study reported here builds on this understanding and clarifies the possible effects of viral influence on apoptosis and cellular survival.

In response to viral infection, the affected cells use pattern recognition receptors (PRR) to recognize viral pathogen-associated molecular patterns, which triggers the production of type I interferons (IFNs). These receptors, including transmembrane Toll-like receptors and cytosolic RIG-I-like receptors, activate downstream interferon regulatory factor 3 (IRF3) and interferon regulatory factor 7 (IRF7), and triggering pathways that cause the production of IFN^16,17^. The N protein is linked to an unbalanced innate immune response in COVID-19-positive individuals, which is typified by a high level of pro-inflammatory cytokines and a weaker and delayed IFN response. The virulence of the virus is enhanced by dysregulation in IFN response, which can cause different serious diseases^18^. By disrupting the cell cycle and obstructing RIG-I signaling, the N protein also reduces the production of IFN-β^11^. Furthermore, the N protein may have an IFN antagonistic function as it is related to the suppression of IFN signaling^19^.

The aggregation of hyperactive immune cells due to an uncontrolled immune reaction is called a cytokine storm, an activation cascade delivered by the synthesis of auto-amplifying cytokines and chemokines. Numerous immune cells of various sorts, epithelial cells, fibrocytes, and cytokines can all be involved in the cytokine storm process. It has been observed that individuals with COVID-19 have expanded levels of some cytokines, consisting of interleukin-9 (IL-9), interleukin-15 (IL-15), interleukin-16 (IL-16), interleukin-18 (IL-18), interleukin-1α (IL-1α), and tumor necrosis factor alpha (TNF-α)^20^. Furthermore, the N protein of SARS-CoV-2 highlights its role in regulating the host's immune response by inducing the production of proinflammatory cytokines, chemokines, and ISGs^21^.

In this study, the SARS-CoV-2 N gene was amplified by a one-step RT-PCR process and inserted into pAdTrack-CMV vector. The immune responses and apoptosis were determined by measuring the interleukin-6 (IL-6), interleukin-12 (IL-12), interleukin-1β (IL-1β), and TNF-α levels, and evaluating the genes expression of BAK, BAX, BCL-2, IRF3, IRF7, and IFN-β.

## Materials and methods

### Amplification of the N gene

Amplification of the N gene (accession number: NC_045512.2) was initiated by employing Allele ID software (version 7.5)^22^ to design primers specifically tailored to the N gene sequence. Two pairs of primers were crafted to obtain the complete N DNA sequence as:

- A forward (N-Fwd) primer with a sequence of: 5′-AAAAGTCGACGCCACCATGTCTGATAATGGACCCCAAA-3′, designed for the N-terminal segment of N gene, which incorporated a SalI site, and.
- A reverse (N-Rev) primer with a sequence of: 5′-AAAAGATATCTTAGGCCTGAGTTGAGTCAG-3′, designed for the C-terminal fragment of N gene, which included an EcoRV site. These sequences were received in lyophilized forms from Metabion (Germany).

To amplify the N gene from the RNA extraction template, we employed the one-step RT-PCR method, utilizing the SuperScriptTM III PlatinumTM one-step RT-PCR kit from Invitrogen (USA). The temperature cycling protocol for the RT-PCR reactions was followed based on the kit instruction including a first crucial step which was selected at 50 °C for 15 min (to facilitate cDNA synthesis within a temperature range of 42–60 °C), followed by denaturation at 95 °C for 2 min, initial denaturation at 95 °C for 15 s, and annealing/extension at 60 °C for 30 s (repeated for a total of 40 cycles). Subsequently, the integrity and size of the PCR product were verified by 1% agarose gel electrophoresis.

### Cloning of N gene in pAdTrack-CMV expression vector

The pAdTrack-CMV vector and PCR product underwent double digestion using SalI and EcoRV enzymes (received from Fermentas, USA) at 37 °C overnight, with each reaction having a final volume of 50 µL and utilizing buffer O (10X). Electrophoresis was then performed to separate the digested products and vectors. Extraction from the agarose gel was performed using a GeneAll kit from GeneAll Biotechnology (Korea). Subsequently, ligation of 100 ng of vector with 3, 5, and 10 ratios of the insert was carried out using the T4 DNA ligase enzyme. The ligated product was then transformed into Top10 competent cells using the heat shock method. The transformed cells were cultured into LB agar supplemented with kanamycin (50 µg mL^−1^) and incubated at 37 °C overnight. The recombinant vector was extracted from several clones using a miniprep kit from Notarkib (Iran).

### Verifying the recombinant vector (pAdTrack-CMV-N) by PCR and sequencing

The extracted recombinant vector underwent PCR with the N-Fwd and N-Rev primers based on the following conditions. Each reaction consisted of 2 μL of the extracted recombinant vector, 0.5 μL of each primer, 10 μL of the master mix (2X), and 7 μL double distilled water resulting in a total reaction volume of 20 μL. The thermal cycling program started with an initial denaturation step at 95 °C for 10 min; then, it went through 30 cycles of denaturation at 95 °C for 30 s, annealing at 60 °C for 30 s, and extension at 72 °C for one minute. Then, a final extension at 72 °C for 10 min was followed. Subsequently, the resulting PCR products were sequenced for analysis. The sequencing results were presented in FASTA file format and imported into the MEGA software. Alignment of the sequences was accomplished using the ClustalW method^23^.

### Cell culture

HEK293 cell line (ATCC CRL-1573) was cultured in a medium composed of the complete Dulbecco's Minimal Eagle Medium, which was further enhanced by adding 10% fetal bovine serum as well as streptomycin sulfate and penicillin for optimal growth conditions. The cells exhibited an adherent growth pattern and were methodically detached using trypsin/EDTA as part of the cultivation process. Throughout the growth, they were maintained in a controlled environment characterized by a humidified atmosphere with 5% CO_2_ concentration at 37 °C.

### Cell transfection with the pAdTrack-CMV-N expression vector

To explore the transient expression of the N gene, we conducted cell transfection within cell culture plates. We utilized the pAdTrack-CMV-N expression vector, along with a control vector (pAdTrack-CMV), to gauge the transfection efficiency employing the polyethyleneimine (PEI, MW 25 kDa) method. To ascertain the expression of the N gene within the cells, we observed the fluorescence of the green fluorescent protein marker by a fluorescent microscope.

### Western blot

For the Western blot analysis, we initiated cell lysis using RIPA buffer from Thermo Fisher Scientific (USA), supplemented with protease inhibitor from Roche (Switzerland). Subsequently, we conducted protein separation through 10% SDS-PAGE, and transferred the separated protein into a polyvinylidene difluoride membrane from Thermo Fisher Scientific (USA). A specific primary antibody was treated with the membrane overnight at 4 °C after they had been pre-blocked. Horseradish peroxidase-conjugated secondary antibody was then added to the membrane for an additional hour at room temperature following the initial incubation period.

### RNA extraction and cDNA synthesis

Extraction of total RNA from HEK293 cells was done by RNA isolation kits from Parstous (Iran). To prevent any DNA contamination, we treated the samples with DNase I enzyme following the kit instruction. A NanoDrop-1000 spectrophotometer (USA) was utilized to assess both RNA concentration and purity, while RNA integrity was confirmed by gel electrophoresis. For the reverse transcription reaction, a cDNA synthesis kit from Parstous (Iran) was used with a reaction volume of 20 μL. This reaction included the following components: 1 μg total RNA as a template, 1 μL of RT enzyme (200 U µL^−1^), 1 μL of RNase inhibitor RiboLock (20 U µL^−1^), 1 μL of random hexamer primer, 2 μL of a 10 mmol L^−1^ dNTP mix, and 4 μL of 5X reaction buffer. The samples underwent incubation for 10 min at 25 °C, followed by 60 min at 42 °C, and finally 5 min incubation at 75 °C.

### Design of oligonucleotide sets

As focal points, BAX, BAK, BCL-2, IRF-3, IRF-7, and IFN-β genes, along with an internal reference gene (β-actin), were selected. To acquire the gene sequences, we utilized the NCBI database; subsequently, Allele ID software was employed to formulate the primer sets. The oligonucleotide sequences are displayed in Table 1.

**Table 1:** Primer sequences for BAX, BAK, BCL-2, IRF3, IRF7, IFN-β, and β-actin genes.

Genes	Orientation primer	Sequence (5′ → 3′)
BAX	Forward	F: AGGGTGGCTGGGAAGGC
	Reverse	R: TGAGCGAGGCGGTGAGG
BAK	Forward	F: GCCTACTGACCCAGAGATGG
	Reverse	R: CTCATAGGCGTTGTCTGCTG
BCL-2	Forward	F: ATGTGTGTGGAGACCGTCAA
	Reverse	R: GCCGTACAGTTCCACAAAGG
IRF-3	Forward	F: CGGAAAGAAGTGTTGCGGTTAG
	Reverse	R: TTTGCCATTGGTGTCAGGAGAG
IRF-7	Forward	F: TGCAAGGTGTACTGGGAG
	Reverse	R: TCAAGCTTCTGCTCCAGCTCCATAAG
IFN-β	Forward	F: CAACTTGCTTGGATTCCTACAAAG
	Reverse	R: TATTCAAGCCTCCCATTCAATTG
β-actin	Forward	F: CCTGGCACCCAGCACAAT
	Reverse	R: GCCGATCCACACGGAGATCT

### Analysis of real-time PCR data

For real-time PCR, each reaction consisted of 2 μL of cDNA, 0.5 μL of each primer, and 12 μL of the reaction buffer, resulting in a total reaction volume of 15 μL. SYBR Green I was employed as a reporter dye. The real-time PCR reactions were conducted using the QuantStudio 3 real-time PCR system from Thermo Fisher Scientific (USA). The thermal cycling program started with a denaturation step at 95 °C for 10 min; then it went through 40 cycles of denaturation at 95 °C for 45 s and annealing at 58–62 °C for 35 s, and extension at 72 °C for 45 s.

### Cytokine quantification analysis

We focused on the precise measurement of four crucial proinflammatory cytokines of IL-6, IL-12, IL-1β, and TNF-α. These cytokines play a central role in regulating the immune response and are vital for understanding inflammatory processes. High sensitivity human enzyme-linked immunosorbent assay (ELISA) kits from Pishtazteb (Iran), each designed for a particular cytokine of interest, were used, and their manufacturer's instructions were followed.

### Statistical examinations

The cycle threshold values obtained from the real-time PCR runs were standardized using the Ct normalization algorithm software (http://ctnorm.sums.ac.ir). Subsequently, the data underwent analysis using Microsoft Excel. For comparing the means, T-test was employed by GraphPad Prism. Statistical significance was considered at *P* values less than 0.05.

## Results

### Expression and verification of the SARS-CoV-2 N protein in HEK293 cells

**Figure 1:**
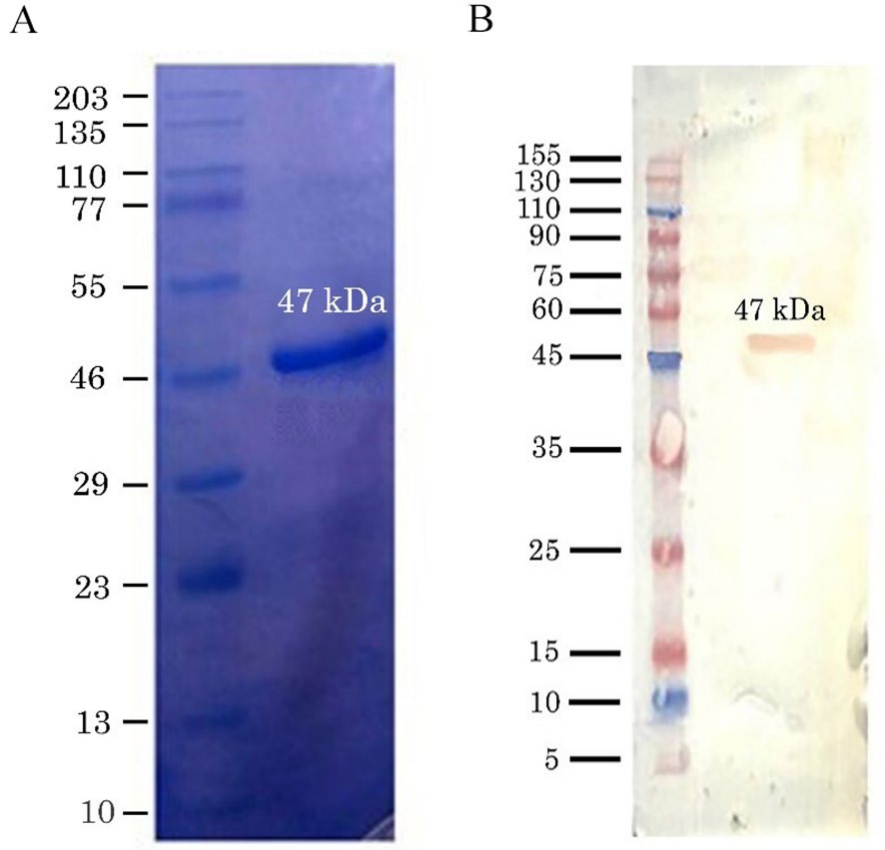
An image from SDS-PAGE gel for the N protein after purification (**A**), and a paper chromatogram of the N protein after binding with specific antibodies (Western blot analysis) (**B**) to confirm the protein purity.

To ascertain the successful expression of the SARS-CoV-2 N gene in HEK293 cells, we followed Western blot procedure. The process commenced with the transfection of the recombinant pAdTrack-CMV-N vector into HEK293 cells. Initially, protein samples underwent separation through SDS-PAGE; then, Western blot was performed. The results of this analysis and verification process are presented in Fig. 1. The results visually confirmed the successful expression of the SARS-CoV-2 N protein in HEK293 cells, providing a crucial foundation for subsequent experiments and investigations related to the protein's function and interactions.

### Effect of SARS-CoV-2 N protein on apoptosis

**Figure 2:**
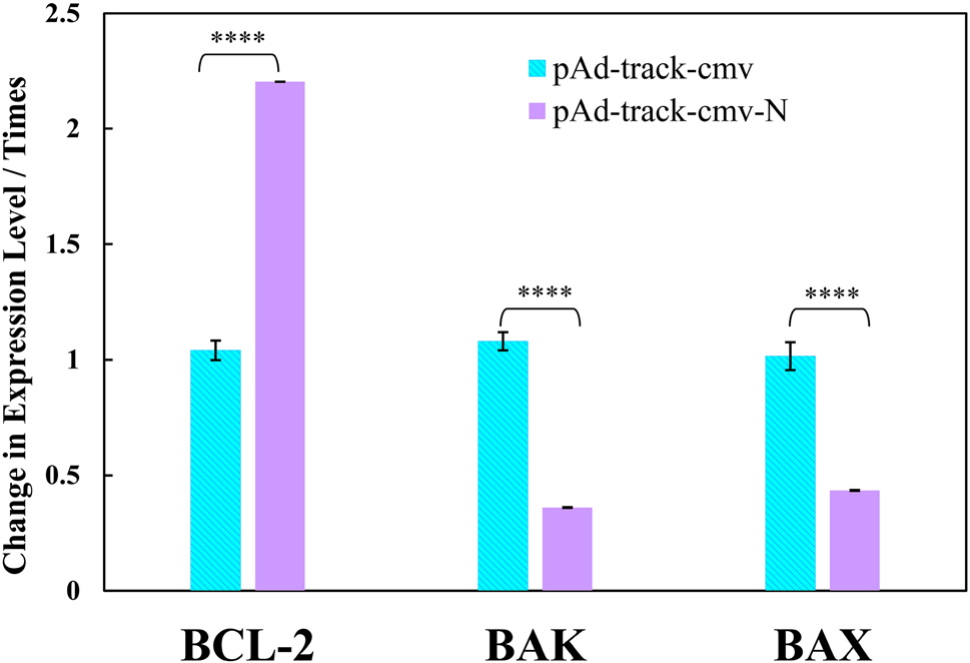
Changes in BCL-2, BAK and BAX genes expression (due to apoptosis) in HEK293 cells treated with (pAdTrack-CMV-N) or without (pAdTrack-CMV) the N gene for 48 h. *P* values were < 0.05.

Significant attention has been paid to the complex mechanisms leading to cell death pathways upon SARS-CoV-2 targeting. However, the specific viral proteins responsible for orchestrating these pathways remain unclear^24^. We investigated the effect of SARS-CoV-2 N protein on apoptosis status of HEK293 cells upon transfecting. To determine the effect of the N protein, we transfected pAdTrack-CMV (empty vector) and pAdTrack-CMV-N into HEK293 cells, and the expression of three genes of BAX, BAK, and BCL-2 mRNAs was evaluated. The pro-apoptotic proteins of BAX and BAK mRNAs, which are crucial in the decision of a cell to undergo apoptosis, were measured. The obtained results are displayed in Fig. 2, indicating a decrement in the expression of the BAX and BAK genes upon pAdTrack-CMV-N, compared to pAdTrack-CMV (empty vector), with significant differences (*P* < 0.05). On the contrary, pAdTrack-CMV-N led to an increment in the expression of the BCL-2 gene, compared to pAdTrack-CMV, with significant differences (*P* < 0.05). These results confirmed the hypothesis regarding apoptosis suppression within HEK293 cells, upon pAdTrack-CMV-N.

### Effect of SARS-CoV-2 N protein on IFN-I

**Figure 3:**
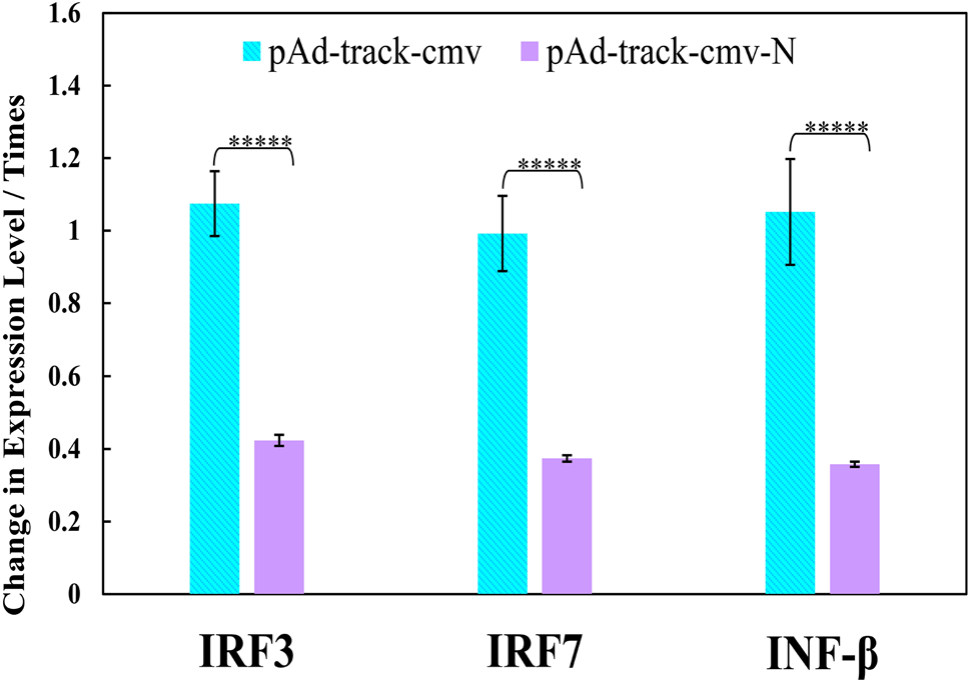
The gene expression levels of IRF3, IRF7, and IFN-β in HEK293 cells after transfection with the empty vector (pAdTrack-CMV) or the N gene vector (pAdTrack-CMV-N). *P* values considered were < 0.05.

To clarify the immune responses of HEK293 cells against viral N protein of SARS-CoV-2, we explored its antagonist effect on the IFN-I pathway to identify the cell antiviral defense. The gene expression levels of three key components affecting the IFN pathway of IRF3, IRF7, and IFN-β were measured, and the results are shown in Fig. 3. The results revealed a substantial decrement in the expression of IRF3, IRF7, and IFN-β genes upon pAdTrack-CMV-N, compared to pAdTrack-CMV (empty vector), with significant differences (*P* < 0.05); this confirms the inhibition of IFN signaling pathway.

### Effect of SARS-CoV-2 N protein on the cytokines level

**Figure 4:**
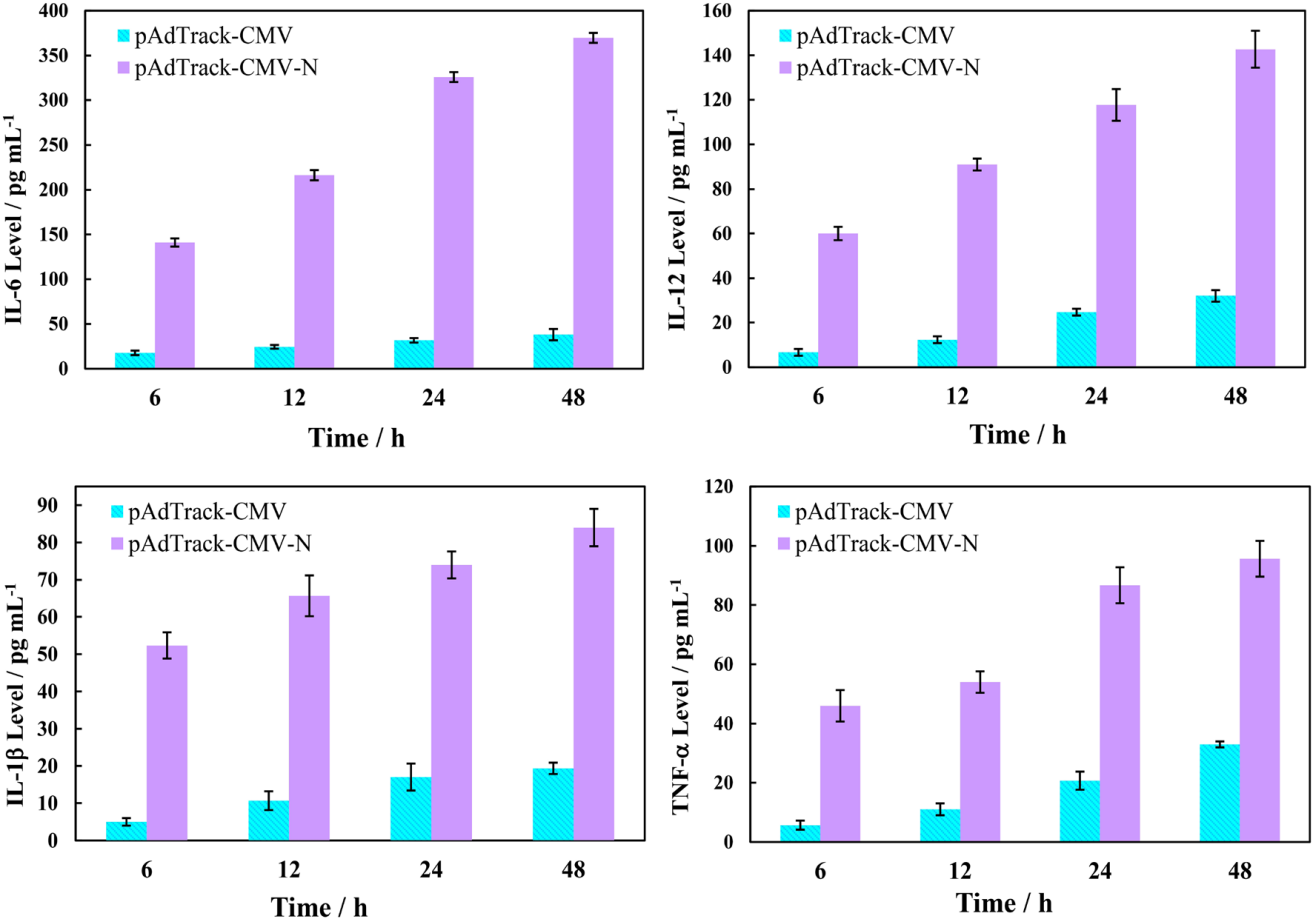
The levels of IL-6, IL-12, IL-1β, and TNF-α in HEK293 cells after transfection with the empty vector (pAdTrack-CMV) or the N gene vector (pAdTrack-CMV-N) at four intervals.

The levels of a series of cytokines of IL-6, IL-12, IL-1β, and TNF-α (as key controlling factors of the immune response and inflammation) upon transfection of the pAdTrack-CMV-N vector into HEK293 cells were measured at four intervals. The results are shown in Fig. 4. Based on these findings, the level of all these cytokines remarkably elevated in the transfected cells, with prolonging the time (compared to the corresponding empty vector). This temporal evolution of cytokine production can have a key role in initiating the cytokine storms and inflammation. It has also been reported that the SARS-CoV-2 N protein is an important factor to start cytokine storms and the increase of inflammation^25^.

## Discussion

Infection by SARS-CoV-2 activates the RIG-I-like receptor pathway and starts the expression of IFN-β, which is one of the major mechanisms of the host innate immune defense. However, the N protein has a capacity to inactivate the RIG-I-like receptor and act against the IFN-I pathways by interaction with Helicase domain of RIG-I^9,26^. Furthermore, it has also been established that the N protein is a key contributor to inflammation, and it potentially impacts the onset of cytokine storms in individuals with severe cases of COVID-19^20^. The N protein (and glycoproteins on the SARS-CoV-2 surface) can activate signal transduction pathways by PRR, leading to activation of NF-κB pathway and then induction of pro-inflammatory cytokines^27^. The N protein also has the capacity to inhibit apoptotic pathways and can prolong the survival of the infected cells^28^ by activation of the NF-κB pathway, leading to regulation of the anti-apoptotic BCL-2 gene^29,30^.

The present research has directed into the involvement of the SARS-CoV-2 N protein in and its impact on HEK293 cells death and the pathogenesis of COVID-19. Our results indicated that while the empty vector did not affect BAK, BAX, and BCL-2 expression (with times of change in the expression level of unity), the N structural protein regulated important apoptotic genes of BAK, BAX, and BCL-2; it also had the potential to individually (without going through other proteins of M, S, and E protein functions) hinder apoptosis in HEK293 cells. These finding are in the same line with those reported elsewhere by Pan et al.^28^ implying an anti-apoptotic mechanism for the SARS-CoV-2 N protein. This mechanism was just related to the interactions between the N and (the anti-apoptotic) myeloid cell leukemia-1 proteins, ruling out the role of BCL-2 protein. However, our results revealed that BCL-2 gene was also involved in the apoptotic pathways induced by the N protein. Contrary to our results and those reported by Pan et al., Ren et al. showed that the SARS-CoV-2 N protein had the capability to enhance apoptosis induced by the M protein through reducing the dampened PDK1-PKB/Akt signaling initiated by the M protein^31^. Moreover, it has also been reported that the N protein induces apoptosis elevation and renal cells death in acute kidney injury though a Smad3-p21-dependent G1 cell cycle arrest mechanism leading to inhibition of tubular epithelial cells proliferation^32^.

Coronaviruses including SARS-CoV-2 virus diminish the host's defense systems, thereby enhancing their capacity to replicate and spread in response to innate immune-mediated viral clearance mechanisms^33,34^. This goes through suppression of IRF3, IRF7, and IFN-β to help it evade the host's immune response^35–37^. SARS-CoV-2 effectively delays the start of the antiviral response by blocking IFN-β production and giving the virus a chance to replicate and spread^13^. In addition, the virus stops the host cell from identifying and reacting to the viral infection by suppressing IRF3 and IRF7 activation. This strategic approach is crucial for the virus to efficiently spread throughout the host organism^38^. Our results showed that while empty vector did not affect IRF3, IRF7, and IFN-β expression (with times of change in the expression level of near unity), transfection of the N protein into HEK293 cells led to decrement in IRF3, IRF7 and IFN-β levels. Similar results for IRF3 and IFN-β levels have been reported previously^11,39^. The N protein binds the SPRY domain of TRIM25 (as an E3 ubiquitin ligase enzyme regulates innate immune response) leading to disruption in TRIM25's ubiquitinating action towards RIG-I and suppresses the production of IFN-β^40^. In addition, interaction between RIG-I and the N protein is facilitated by RIG-I's DExD/H domain, leading to suppression of IRF3 phosphorylation and subsequent translocation into the cell nucleus^11^. Suppressing activation of antiviral IRFs (such as IRF3 and IRF1) by SARS-CoV-2 has been reported^41,42^. On the other hand, SARS-CoV-2 proteins of nsp13, nsp14, nsp15, and orf6 operate as inhibitors of IFN signaling pathways (and reduce the expression and synthesis of IFN gene)^33,37^. Our results revealed the N protein function in suppressing the IRF7 level in virus-infected cells. It has been shown that inhibition of the function of TANK-binding kinase 1 (TBK1) on IRF3 by the N protein results in suppression of IRF3 phosphorylation and activation. On the other hand, interaction of the N protein with TBK1 has been confirmed^39^. Therefore, it can be deduced that the N protein suppresses the IRF7 phosphorylation (and activation) and ultimately nuclear translocation. Likewise, the N protein can interact with RIG-I^11^ to alter the TBK1 function^43^.

Several studies have shown that cytokine levels rise after infection with SARS-CoV-2. Based on the Hasan et al.’s study, IL-6 production was enhanced in patient sera upon incubation with a commercially available N protein^44^. According to Wang et al., lung epithelial A549 cells released different cytokines in response to the N protein, rather than the S protein^20^. The levels of IFN-γ, IL-8, IL-6, and IL-1 cytokines have also been related to mutations in the N protein^21^. Our results showed that the empty vector induced gentle increments in the levels of IL-6, IL-12, IL-1β, and TNF-α cytokines over time. This is due to the experimental manipulation of the transfection of the empty vector. Such system stress leads to an increment in the cytokines level^45^. On the other hand, the N protein transfection induces a huge rise in the levels of IL-6, IL-12, IL-1β, and TNF-α cytokines. It should be noted that increments in the cytokine levels upon the protein transfection in this study were much higher than other related research (compared to the corresponding controls)^20,44^.

## Conclusion

This study uncovers crucial insights about the mechanisms involved in the SARS-CoV-2, offering new knowledge into interactions between SARS-CoV-2 and host cells. The N protein of SARS-CoV-2 potentially influences the genes associated with cell death, promoting an anti-apoptotic effect that extends the survival of the infected cells. This finding has been confirmed by a few researchers. The N protein plays a significant role as an antagonist of the IFN-I through a decrease in IRF3, IRF7, and IFN-β genes expression. On the other hand, the N protein plays a significant role as a promoter of the pro-inflammatory cytokines of IL-6, IL-12, IL-1B, and TNF-α production through excessive increase in the corresponding genes expression. The findings seem to be essential for developing targeted treatments against COVID-19 and would be helpful to prevent negative side effects of the cytokine storm. Further research in this field may reveal additional strategies to effectively face this viral threat.

## Data Availability

All the data and materials related to this study are presented in the paper text, table and figures.

## Abbreviations

COVID-19: Coronavirus disease 2019
SARS-CoV-2: Severe acute respiratory syndrome coronavirus 2
ORFs: Open reading frames
NSP: Non-structural protein
S: Spike
E: Envelope
M: Membrane
N: Nucleocapsid
ACE2: Angiotensin-converting enzyme-2
IFN-β: Interferon beta
RIG-I: Retinoic acid-inducible gene I
MAVS: Mitochondrial antiviral-signaling
BCL-2: B-cell lymphoma-2
BAK: BCL-2 antagonist/killer
BAX: BCL-2 antagonist X
HEK293: Human embryonic kidney 293
PRR: Pattern recognition receptors
IFNs: Interferons
IRF3: Interferon regulatory factor 3
IRF7: Interferon regulatory factor 7
IL-9: Interleukin-9
IL-16: Interleukin-16
IL-18: Interleukin-18
IL-1α: Interleukin-1α
IL-15: Interleukin-15
TNF-α: Tumor necrosis factor alpha
IL-6: Interleukin-6
IL-12: Interleukin-12
IL-1β: Interleukin-1β
ELISA: Enzyme-linked immunosorbent assay
TBK1: TANK-binding kinase 1
